# Membrane Orientation and Binding Determinants of G Protein-Coupled Receptor Kinase 5 as Assessed by Combined Vibrational Spectroscopic Studies

**DOI:** 10.1371/journal.pone.0082072

**Published:** 2013-11-22

**Authors:** Pei Yang, Alisa Glukhova, John J. G. Tesmer, Zhan Chen

**Affiliations:** 1 Department of Chemistry, University of Michigan, Ann Arbor, Michigan, United States of America; 2 Departments of Pharmacology and Biological Chemistry, Life Sciences Institute, University of Michigan, Ann Arbor, Michigan, United States of America; 3 Program in Chemical Biology, University of Michigan, Ann Arbor, Michigan, United States of America; University of Akron, United States of America

## Abstract

G-protein coupled receptors (GPCRs) are integral membrane proteins involved in a wide variety of biological processes in eukaryotic cells, and are targeted by a large fraction of marketed drugs. GPCR kinases (GRKs) play important roles in feedback regulation of GPCRs, such as of β-adrenergic receptors in the heart, where GRK2 and GRK5 are the major isoforms expressed. Membrane targeting is essential for GRK function in cells. Whereas GRK2 is recruited to the membrane by heterotrimeric Gβγ subunits, the mechanism of membrane binding by GRK5 is not fully understood. It has been proposed that GRK5 is constitutively associated with membranes through elements located at its N-terminus, its C-terminus, or both. The membrane orientation of GRK5 is also a matter of speculation. In this work, we combined sum frequency generation (SFG) vibrational spectroscopy and attenuated total reflectance-Fourier transform infrared spectroscopy (ATR-FTIR) to help determine the membrane orientation of GRK5 and a C-terminally truncated mutant (GRK5_1-531_) on membrane lipid bilayers. It was found that GRK5 and GRK5_1-531_ adopt a similar orientation on model cell membranes in the presence of PIP_2_ that is similar to that predicted for GRK2 in prior studies. Mutation of the N-terminal membrane binding site of GRK5 did not eliminate membrane binding, but prevented observation of this discrete orientation. The C-terminus of GRK5 does not have substantial impact on either membrane binding or orientation in this model system. Thus, the C-terminus of GRK5 may drive membrane binding in cells via interactions with other proteins at the plasma membrane or bind in an unstructured manner to negatively charged membranes.

## Introduction

 The duration of signaling by most G-protein coupled receptors (GPCRs) is regulated by the activity of GPCR kinases (GRKs), which phosphorylate the third cytoplasmic loop or C-terminal tails of activated receptors and thereby initiate their uncoupling from heterotrimeric G proteins and subsequent downregulation [1]. There are three GRK subfamilies, represented by GRK1, GRK2, and GRK4 [2]. The three subfamilies have distinct mechanisms by which they associate with membrane lipid bilayers. GRK1, expressed in rod cells, is recruited in a light-dependent manner by meta-rhodopsin with the assistance of its C-terminal farnesyl modification [3]. GRK2 is recruited to membranes via the interaction of its pleckstrin homology (PH) domain with Gβγ subunits [4]. Its membrane orientation is expected to be similar to that which would be predicted from examining the structure of the GRK2-Gβγ complex if one juxtaposed the expected membrane binding surfaces of its PH domain and Gβγ with the membrane [5]. GRK4 subfamily members (GRK4, GRK5, and GRK6) bind to negatively charged lipid bilayers, and the molecular determinants responsible have been mapped to a highly positively charged amino acid region close to the N-terminus believed to bind PIP_2_ and other anionic phospholipids (GRK5 residues 22-29) [6,7] and/or a basic amphipathic helix close to its C-terminus (GRK5 residues 546-565) [7,8]. Finally, palmitoylation sites are found immediately C-terminal to the amphipathic helix in GRK4 and in the GRK6A splice variant [9,10], thereby reinforcing the idea that this region is involved in membrane binding. However, palmitoylation does not occur in GRK5 and thus is not required for membrane binding or the function of this enzyme [11-14]. Consequently, it is not clear if GRK4 subfamily members associate with membranes in an orientation similar to that predicted for GRK2, or whether a specific orientation is induced only in the presence of certain anionic phospholipids and/or by activated receptors. However, because of their homology and the fact that they phosphorylate many GPCRs with similar catalytic efficiency, it is anticipated that the membrane-bound orientations of all GRKs are similar.

Recently, the role of C-terminal amphipathic helix of GRK4 subfamily members was brought into question by the structure of GRK6 in what is expected to be a relatively active conformation, wherein the helix docks to the catalytic core of the enzyme at a site ~30 Å distant from the N-terminal basic region, where sulfate anions were observed to bind [15]. This C-terminal region was disordered in a prior structure of GRK6 wherein the kinase domain adopts an inactive conformation [16]. Therefore, it is possible that the C-terminal helix only interacts with the membrane when the kinase is inactive, and assumes a structural role when the kinase adopts an active configuration. Such would be consistent with the observation that mutation of residues within the C-terminal helix inhibits phosphorylation of the soluble substrate tubulin in the absence of phospholipid vesicles [8].

Although the membrane-bound orientation of proteins such as GRK5 is relevant to many biological questions [17-22], it is difficult to determine this orientation in a biologically relevant environment. Recently, we demonstrated that combined sum frequency generation (SFG) vibrational spectroscopy and attenuated total reflectance-Fourier transform infrared spectroscopy (ATR-FTIR) can be combined to more accurately determine the interfacial orientation of complicated protein molecules [23-30]. SFG is a surface-sensitive nonlinear optical vibrational spectroscopic technique that can provide *in situ* structural information about molecules at interfaces [31-45], and SFG has been successfully applied to examine interfacial peptides/proteins at the molecular level [46-58]. ATR-FTIR spectroscopy has also been used to study peptide/protein orientation at interfaces [59-63]. The combination of SFG and ATR-FTIR provides more independent parameters that can be used to define the orientation of interfacial molecules [25,64-69]. In our previous research, we successfully developed a methodology to apply the combined vibrational spectroscopic approach to determine membrane orientation of G-proteins *in situ* based on the overall orientation of α-helical components in the molecule [25].

To determine the orientation of a GRK4 subfamily member at the membrane and assess how this orientation is influenced by the C-terminal amphipathic helix, combined SFG and ATR-FTIR analysis was used to determine the orientation of GRK5 (residues 1-590) and a variant truncated after residue 531 (GRK5_1-531_) on negatively charged lipid bilayers containing phosphatidyl glycerol or phosphatidyl inositol -4,5-bisphosphate. The behavior of a variant with mutations in its N-terminal basic region (GRK5_NT_) was also tested. Because the crystal structure of GRK5 is not available, we used two different crystal structures of its close homolog GRK6 to deduce the membrane orientation for each GRK5 variant. The inactive conformation of GRK6 (PDB entry 2ACX) seems to satisfy the data analysis for GRK5 orientation determination better than its closed conformation (PDB entry 3NYN), consistent with the fact that no receptor is present in the preparation and 3NYN is thought to resemble a receptor-bound conformation. The similarity of the resulting orientations suggests that the interaction between GRK5 and GRK5_1-531_ with negatively charged lipid bilayers are mainly due to the N-terminal basic region. The presence of a C-terminal amphipathic helix in GRK5 does not have substantial impact on membrane binding or orientation under our experimental conditions, but this region may still help tether the enzyme to lipid bilayers in an unstructured manner.

## Materials and Methods

### Protein Samples

GRK5, GRK5_1-531_ and GRK5-K26/28/29/31/35A (GRK5_NT_), which has 3 mutations in common with the PIP_2_-binding deficient GRK5_NT_ variant described in Ref. 6, and 2 additional mutations extending into calmodulin binding site [70,71], were expressed in High Five insect cells using Bac-to-Bac system (Invitrogen). The GRK5 variants were all purified similarly to that described for GRK1 [72]. Cells were lysed in 20 mM HEPES pH 8.0, 300 mM NaCl, and 10 mM β-mercaptoethanol, containing 0.5 mM EDTA, 1 mM PMSF, 3 mg/l of leupeptin and 3 mg/l of Lima Bean inhibitor using a C3 Avestin homogenizer. After ultracentrifugation the soluble fraction was applied to the Ni-NTA column, equilibrated with 20 mM HEPES pH 8.0, 300 mM NaCl and 10 mM β-mercaptoethanol. The column was washed with equilibration buffer supplemented with 10 mM imidazole, followed by elution with equilibration buffer containing 200 mM imidazole. Fractions containing GRK5 were pooled together, diluted to less than 50 mM NaCl with 20 mM HEPES pH 8.0, and 2 mM DTT and applied to Source 15S column (Amersham Biosciences) pre-equilibrated in buffer A (20 mM HEPES pH 8.0, 50 mM NaCl and 2 mM DTT). Elution was performed using a salt gradient from 50 to 500 mM in buffer A. Fractions were analyzed by SDS-PAGE and Coomassie staining. Samples containing GRK5 were pooled together, concentrated and frozen in liquid nitrogen until further use. On the day of the experiment, GRK5 samples were thawed and buffer exchanged into 20 mM HEPES pH 8.0, 50 mM NaCl, and 2 mM DTT using fast desalting Micro Bio-Spin 6 column (Bio-Rad). This buffer was also used as the liquid subphase for the lipid bilayer in SFG and ATR-FTIR experiments.

### SFG Spectroscopy

SFG spectroscopy is a process in which two input beams at frequencies *ω*_*vis*_ and *ω*_*IR*_ mix in a medium and generate an output beam at the sum frequency *ω*_*sum*_=*ω*_*vis*_+*ω*_*IR*_. This is a second-order nonlinear optical process, and transitions are only allowed in media without inversion symmetry. On surfaces and at interfaces where this inversion symmetry is broken, SFG signals can be generated. SFG technique has been successfully used to measure the interfacial orientation of a variety of peptides and proteins in model cell membranes [64-69]. Such orientation information can be deduced by the intensity ratio of SFG spectra collected using different polarization combinations of the input and output laser beams. Relevant theoretical background and the design of our SFG spectrometer are described elsewhere [64-69]. In this research, all SFG experiments were carried out at room temperature (24°C). SFG spectra from GRK5 with different polarization combinations including ssp (s-polarized SF output, s-polarized visible input, and p-polarized infrared input) and ppp were collected using near total internal reflection geometry [65-68].

Planar supported lipid bilayers (PSLBs) were used as model cell membranes in this study. They were prepared on clean right-angle CaF_2_ prisms (Altos Photonics) by using the Langmuir-Blodgett/Langmuir Schaefer (LB/LS) method, as described previously [26]. In order to mimic the cell membrane environment, 9:1 and 1:1 mixtures of 1-palmitoyl-2-oleoyl-sn-glycero-3-phosphocholine (POPC) and 1-palmitoyl-2-oleoyl-sn-glycero-3-phosphoglycerol (POPG) lipids were used to make lipid bilayers. Pure POPG lipid bilayers were also used in our experiment as well as a 1:1 mixture of POPC and 1,2-dioleoyl-*sn*-glycero-3-phospho-(1'-myo-inositol-4',5'-bisphosphate) (PIP_2_). Lipids were purchased from Avanti Polar Lipids Inc., dissolved in chloroform, and mixed as needed to produce the desired lipid mixture composition.

**Figure 1:**
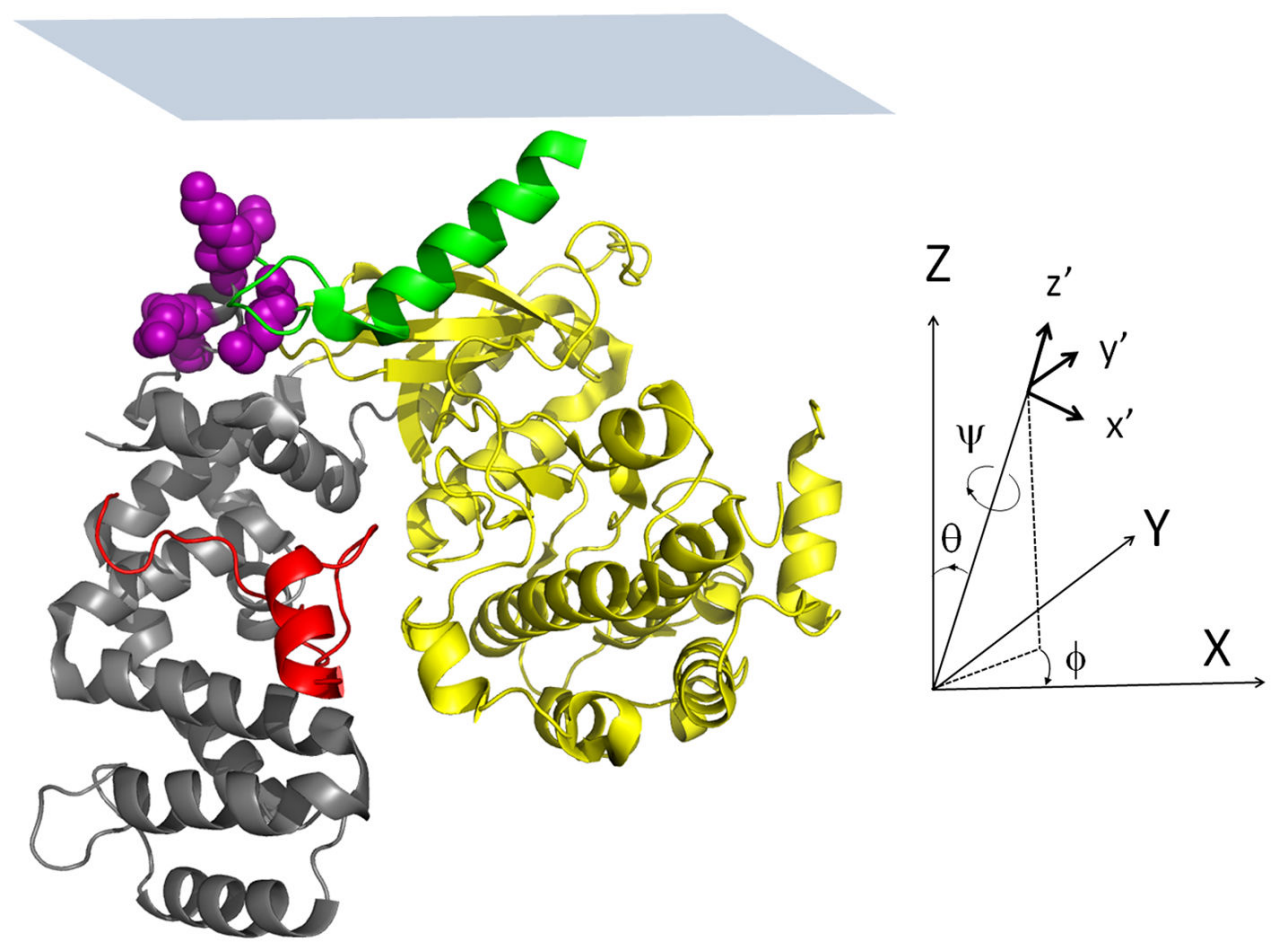
GRK5 in the reference orientation. The GRK5 (PDB entry: 3NYN) and definition of twist (ψ), tilt (θ) and azimuthal (ϕ) angles which rotate the protein from the molecular (x´, y´, z´) to the macroscopic (X, Y, Z) coordinate system. The regulator of G protein signaling homology (RH) domain is colored grey, the C-terminal region containing the amphipathic helix is colored red, the kinase domain yellow, and the αN helix green (18). The side chains of residues proposed to be involved in the N-terminal phospholipid binding site (K26A, K28A, K29A, K31A, K35A) are shown as purple spheres. An approximate membrane plane (defined to be consistent with Ref. 18), is shown as blue rectangle, and lies parallel to the X-Y plane. The GRK5 is depicted in the reference orientation (ψ=0°, θ=0°, ϕ=0°) used as a starting point for data analysis. In our calculation, the molecule is rotated using an Eulerian rotation scheme according to three angles: first twist (ψ) then tilt (θ) and finally azimuthal (ϕ).

Following the equilibration of the lipid bilayers, the aqueous subphase was flushed three times with fresh buffer to remove excess lipids. GRK stock solutions were then injected into the aqueous subphase to a final concentration of 336 nM, and allowed to reach equilibrium for 1 h. SFG spectral intensity at 1655 cm^-1^ was monitored for 1 h as the system was allowed to equilibrate, after which time SFG spectra from the membrane associated GRK5 variants were collected in the ppp and ssp polarization combinations, allowing us to probe different components of the second order nonlinear optical susceptibility tensor [5,26]. As described previously, the experimentally measured tensor components can then be used to determine the membrane-bound orientation by calculating the molecular response from a single protein [5,25]. We developed a computer program that can perform these calculations in a semi-automated fashion for proteins based on the α-helical segments within the protein [5]. By comparing the results of this program with the experimental SFG polarized spectra makes it possible to characterize the orientation of membrane-bound protein molecules in terms of the twist (ψ) and tilt (θ) angles relative to an arbitrary reference position [5,25]. The rotations corresponding to these angles are shown in Figure 1. The reference orientation of GRK5 was chosen to be the same as that for GRK1 in Ref. 18.

### ATR-FTIR Spectroscopy

For ATR-FTIR measurements, a total internal reflection scheme was used to produce surface sensitivity on the order of hundreds of nanometers to microns by controlling the penetration depth of the evanescent wave into the sample. POPG, 9:1 POPC:POPG, and 1:1 POPC:PIP_2_ mixed lipid bilayers were prepared on clean ZnSe substrates (Specac, UK) for ATR-FTIR measurement by using the LB/LS method, as in the SFG experiment. For the ATR-FTIR experiments, deuterated buffer solutions were used in order to avoid spectral interference from water bending signals that would otherwise overlap with the protein amide I signals of interest. Protein samples in deuterated solvent were injected into the subphase for a target concentration of 336 nM to match the concentrations we used in SFG experiments, and samples were allowed to equilibrate for 2 h prior to data collection.

ATR-FTIR spectra were collected in the p and s polarizations on a Nicolet 6700 spectrometer with the ATR-FTIR accessory. Unpolarized ATR-FTIR spectra were also collected and used to compare either the surface coverage of GRK samples on different lipid bilayers (pure POPG bilayer vs. 9:1 POPC:POPG mixed bilayer) or different GRK samples on the same lipid bilayers (GRK5 vs. GRK5_NT_ on 1:1 POPC:PIP_2_). All ATR-FTIR spectra presented here are the average of 128 scans. In order to reduce interference from water vapor present in the air, the instrument was purged with dry N_2_ prior to use, and spectra were afterwards corrected for trace amounts of water vapor using an additional background correction based on the spectrum of pure water vapor in air at 24 °C [25].

The background subtraction and a baseline correction in the amide I region were performed in OMNIC 8.3, then fit to a Gaussian line shape using a nonlinear curve fitting algorithm in Origin 8.1. The dichroic ratio R^ATR^ was determined from the ratio of the fitted signal strength in the p and s polarizations of the infrared beam. Using different polarizations of the incident infrared radiation, ATR-FTIR can be used to measure the orientation or order parameters of molecules by relating orientation to a parameter known as the dichroic ratio R^ATR^ [25]. We have adapted this method to determine membrane orientation of large proteins based on the data from their α-helical segments [25]. 

### Combined SFG and ATR-FTIR studies on membrane protein orientation

We have previously determined the membrane orientation of heterotrimeric G proteins using a combination of SFG and ATR-FTIR [25]. In most cases, SFG measurement alone is not enough to determine a single unique orientation, and therefore we developed a software package, similar to that described for SFG above, for ATR-FTIR measurements [25]. Because SFG and ATR-FTIR are both the surface sensitive techniques and measure different orientation parameters, they provide independent results, and the final deduced possible range of protein membrane orientation should satisfy both the SFG and ATR-FTIR measurements, and their combination yields a much narrower range of possible membrane orientations of the protein sample. In order to account for the possible errors in the experiments and data analysis, heat maps were used to display the final possible protein orientation range (different colors represent the quality of match), as discussed in our previous publication [25].

## Results

### Orientation of GRK5 variants

**Figure 2:**
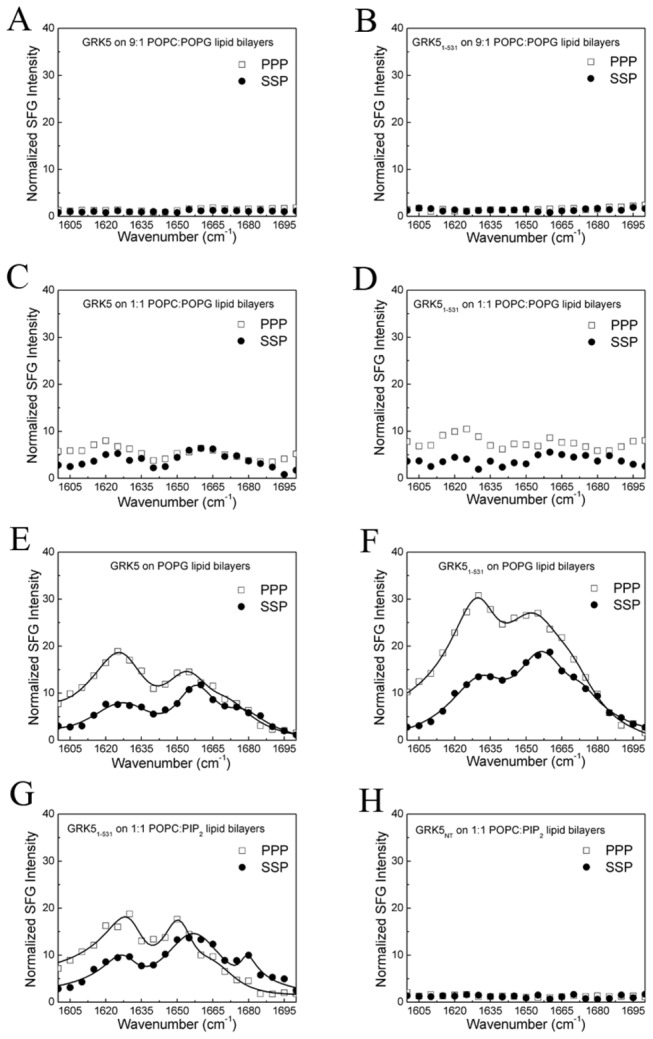
SFG signal of GRK5 and GRK5_1-531_. No discernible SFG amide I signals were observed for 336 nM (A) GRK5 and (B) GRK5_1-531_ interacting with a 9:1 POPC:POPG lipid bilayer. SFG polarized amide I signals of 336 nM (C) GRK5 and (D) GRK5_1-531_ interacting with a 1:1 POPC:POPG lipid bilayer. SFG polarized amide I signals of 336 nM (E) GRK5 and (F) GRK5_1-531_ interacting with a POPG lipid bilayer. SFG polarized amide I signal of 336 nM (G) GRK5_1-531_ and (F) GRK5_NT_ interacting with a 1:1 POPC:PIP_2_ lipid bilayer. The circles and squares are experimental data. The solid lines indicate the fitting results.

We opted to use GRK5 for our studies instead of GRK6 because prior studies indicated that GRK5 is constitutively membrane bound and the degree of its palmitoylation upon expression in insect cells is difficult to assess. We first attempted to determine the possible membrane orientations of full length bovine GRK5 associated with a negatively charged lipid bilayer. We began by collecting SFG amide I signal on 9:1 POPC:POPG lipid bilayers that were previously used for orientation analysis of Gβ_1_γ_2_, GRK2-Gβ_1_γ_2_ and Gα_i_β_1_γ_2_ [5,25]. However, no SFG amide I signal could be detected from GRK5 (Figure 2A). This result either indicates that GRK5 does not bind to these bilayers, or that when bound to the bilayer it exhibits a random distribution of orientations such that SFG signals from its α–helical components cancel out. However, because we could detect the unpolarized ATR-FTIR signals from GRK5 (Figure S1 in File S1), it appears that GRK5 adopts a random orientation when bound to these bilayers.

We then increased the negative charge in the model membranes by using 1:1 POPC:POPG, which we hypothesized would drive GRK5 into more ordered orientation due to interactions with either of its N- or C-terminal basic regions. Weak amide I SFG signals were detected (Figure 2C), but the detected SFG signals were still too weak to perform orientation analysis. We increased the negative charge of the lipid bilayer once again by using a pure POPG lipid bilayer, which produced much stronger SFG amide I signal intensities that could be reliably fit. As shown in Figure 2E, three peaks were observed at the amide I frequency range. The peaks at 1631 and 1671 cm^-1^ are from β-sheet structure, whereas the peak centered at 1656 cm^-1^ is from α-helical structure [5,25,65]. After fitting the SFG ssp and ppp spectra shown in Figure 2E and deconvoluting the Fresnel coefficients for the two polarizations, it was found that the measured ratio of $\chi_{zzz}^{\left(2\right)}/\chi_{xxz}^{\left(2\right)}$ for the GRK5 α-helical structure at the peak center of 1656 cm^-1^ was 0.93.

**Figure 3:**
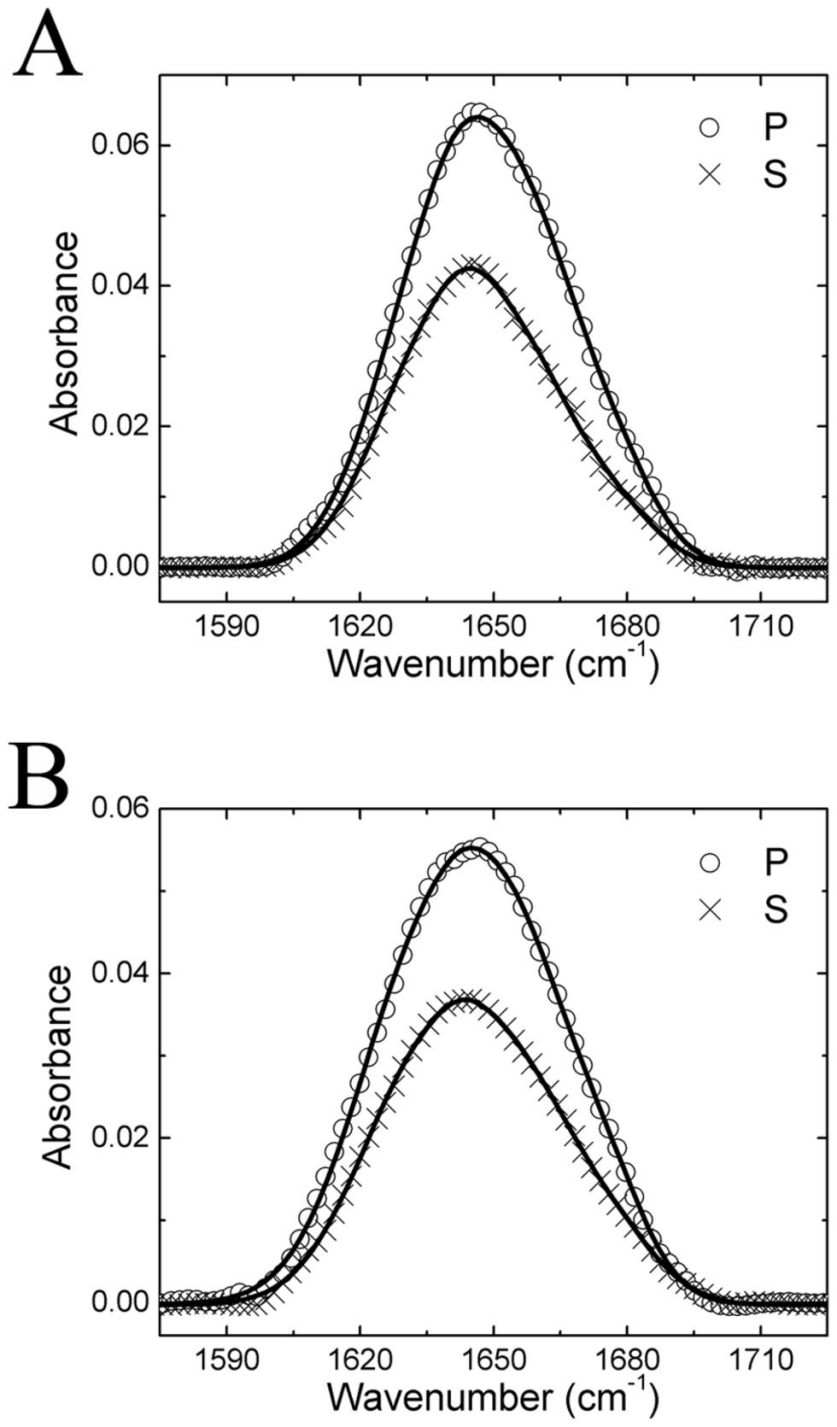
ATR-FTIR spectra of GRK5 and GRK5_1-531_. Experimental ATR-FTIR spectra of 336 nM (A) GRK5 and (B) GRK5_1-531_ on POPG lipid bilayers for the p and s polarizations. The circles and crosses are experimental data. The solid lines are fitting results.

ATR-FTIR spectra of GRK5 on POPG lipid bilayers were then measured (Figure 3A). Four vibrational peaks were evident. The peak centers of the signals contributed by the β-sheet structure were at 1635 and 1670 cm^-1^. The signal at 1643 cm^-1^ is from the random coil/disordered structure, and that at 1658 cm^-1^ is from α-helical structure [25]. After fitting the ATR-FTIR spectra, we calculated a dichroic ratio R^ATR^=1.50 for the peak at 1658 cm^-1^.

The crystal structure of GRK5 is not available, however its sequence is over 70% identical to human GRK6A, which has been crystallized in two different states: a more active conformation with well-ordered N- and C-termini (PDB entry 3NYN) and an inactive conformation with disordered N- and C-termini where the electron density could be reliably modeled only for residues 24-532 (vs. 1-557 residues for 3NYN) (PDB entry 2ACX). The kinase domains adopt distinct conformations in these two structures, changing the relative orientation of many of its α-helical elements. We used both 3NYN and 2ACX crystal structures in the data analysis in order to determine which gave a better match for the GRK5 orientation determination. Figure 1 shows the reference orientation of the 3NYN structure, where the N-terminus is in close proximity with the lipid bilayer and the C-terminus is colored in red [18]. This orientation places its PIP_2_ binding site and its N-terminal helix (αN), which has been proposed to be the primary GPCR binding determinant [19], in close proximity with the membrane plane (colored in green). Assuming that the protein conformation does not dramatically change upon binding to the membrane, we can determine the possible twist (ψ) and tilt (θ) angles of GRK5 by combining the independent SFG and ATR-FTIR experimental measurements and comparing them to those calculated from the two atomic models of GRK6 [5,25]. The most likely membrane orientations of the protein are those that correspond to the orientation range that satisfies both SFG and ATR-FTIR measurements.

**Figure 4:**
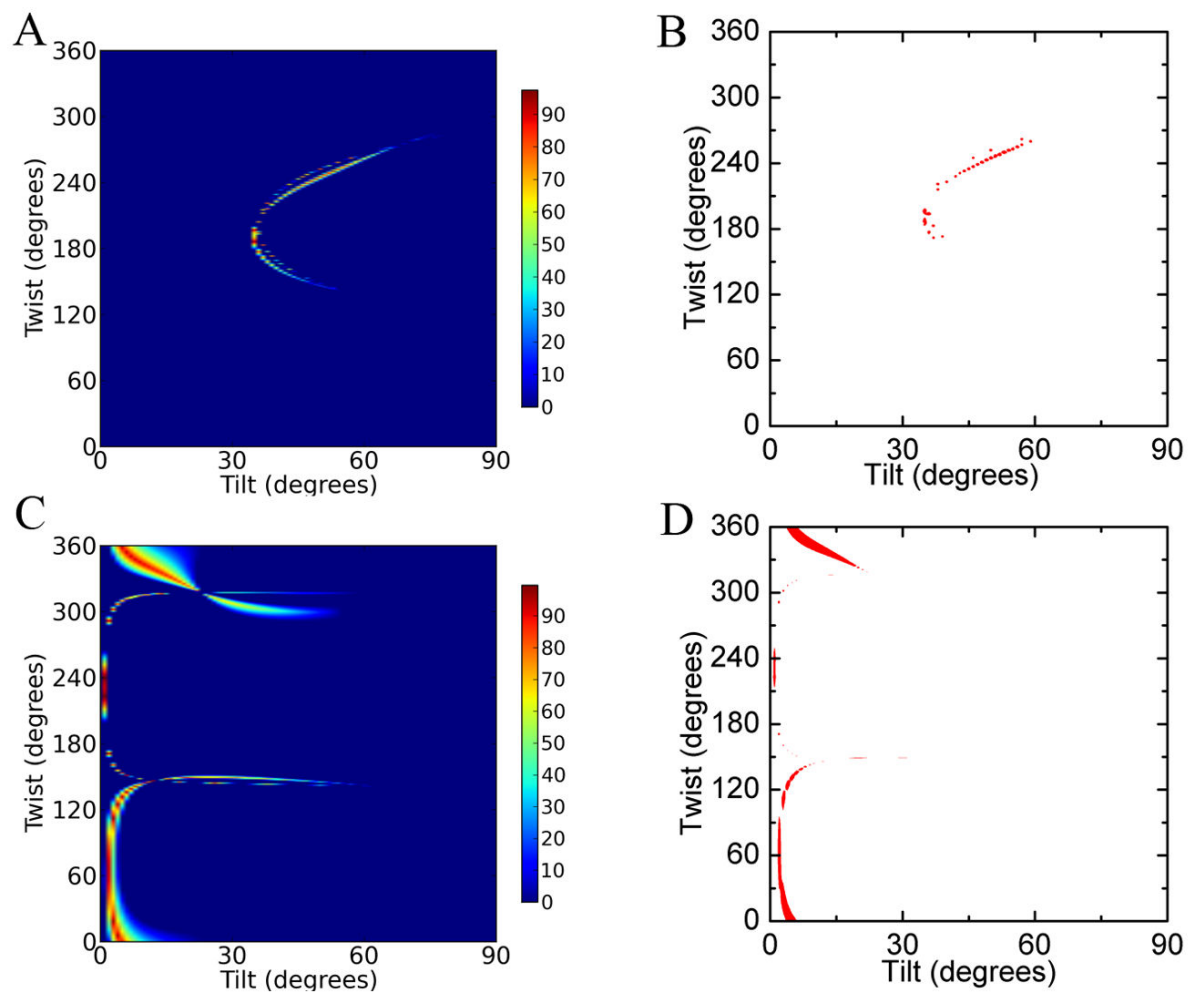
Possible orientations of full length GRK5 by using 3NYN or 2ACX crystal structures. (A) The determined possible orientations of GRK5 on POPG lipid bilayers by combination of SFG and ATR-FTIR measurements (Figure S2 in File S1) by using the 3NYN crystal structure. The effect of experimental errors (such as uncertainty in the Fresnel coefficients) is accounted for using a coloring scheme based on how well the calculated and experimentally measured quantities agree for each possible orientation. The total score is calculated as the product of the scores for all individual criteria. A score of 100% indicates an exact match for all experimental measurements. (B) The same plot as panel A, but only showing orientation areas with a score ≥ 70% (red). (C) The possible orientations of GRK5 on POPG lipid bilayers determined by combination of SFG and ATR-FTIR measurements (Figure S3 in File S1) using the crystal structure of 2ACX. (D) The same plot as panel C, but only showing orientation areas with a score ≥ 70% (red).

**Figure 5:**
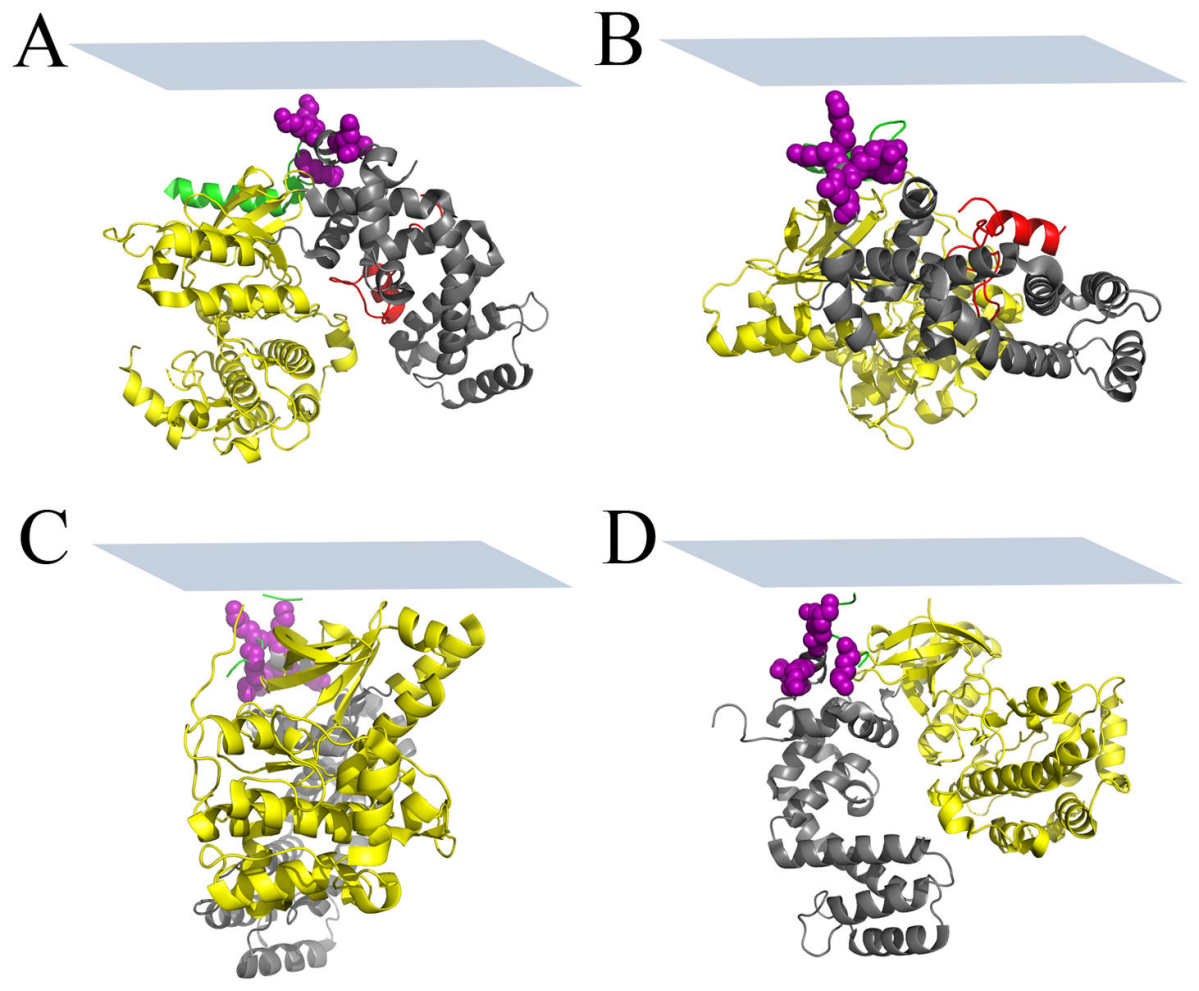
Modeled membrane orientations of full length GRK5. Possible membrane orientations of GRK5 on POPG lipid bilayers as determined from SFG and ATR-FTIR experimental measurements using the 3NYN crystal structure: (A) twist=190°, tilt=35°, (B) twist=245°, tilt=50°. Possible membrane orientations of GRK5 as determined from SFG and ATR-FTIR experimental measurements by using the 2ACX crystal structure: (C) twist=70°, tilt=2°, (D) twist=340°, tilt=10°. The plane of the membrane relative to the protein is shown as a blue rectangle.

Figure 4A and 4B present the possible membrane orientations of GRK5 by combining the SFG and ATR-FTIR measurements using the 3NYN crystal structure. In the heat map (Figure 4A), we use different colors represent the quality of match, and a score of 100% indicates an exact match of experimental measurements [5]. A score ≥70% indicates likely regions. There are two likely regions of (twist, tilt) for GRK5: (170-200°, 35–40°) and (220-260°, 45–60°), as represented in Figure 5A (190°, 35°) and 5B (245°, 50°), and it is clear that they represent two completely different orientations wherein different regions of the protein are juxtaposed with the membrane. Using the 2ACX crystal structure, which adopts a less active conformation, to perform the same orientation analysis yields the orientations shown in Figure 4C and 4D, wherein the most likely orientation regions correspond to (twist, tilt) of (30-150°, 0–10°) and (330-360°, 0-15°). Two orientations of GRK5 deduced from this analysis are shown in Figure 5C (70°, 2°) and 5D (340°, 10°). Comparison with Figure 4B suggests that the 2ACX structure results in a better match with the experimental data as judged by the height of the contour plot. Furthermore, the orientations calculated from the different 2ACX regions are very closely related, with the N-terminal PIP_2_ binding site of GRK6 juxtaposed with the lipid bilayers in each case.

We next examined if truncation of GRK5 after residue 531, which eliminates the C-terminal amphipathic helix and other sequences after the last ordered residue in the 2ACX structure, has an effect on the orientation of GRK5. As shown in Figure 2B, 2D, and 2F, GRK5_1-531_ generates no SFG amide I signal on the 9:1 POPC:POPG mixed bilayer, a very weak SFG signal on the 1:1 POPC:POPG bilayer, and very strong SFG signal on the pure POPG lipid bilayer. These behaviors are similar to those of full-length GRK5 (Figure 2A, 2C and 2E). Unpolarized ATR-FTIR results indicate that GRK5_1-531_ has similar surface coverage on the 9:1 POPC:POPG mixed bilayer and the pure POPG lipid bilayers (Figure S1 in File S1). Therefore the GRK5_1-531_ molecules exhibit a random distribution of orientations on the 9:1 POPC:POPG lipid bilayer such that SFG signals from the α–helical components cancel out (Figure 2B). On the other hand, GRK5_1-531_ molecules adopt a more ordered (or preferred) orientation on the pure POPG lipid bilayer likely due to more negative charge, as strong SFG signals were observed (Figure 2F). On these bilayers, the measured ratio of $\chi_{zzz}^{\left(2\right)}/\chi_{xxz}^{\left(2\right)}$ for the GRK5_1-531_ α-helical structure at the SFG peak center of 1656 cm^-1^ was 1.35 (Figure 2F), and the dichroic ratio R^ATR^ =1.52 (Figure 3B) for the ATR-FTIR peak center of 1658 cm^-1^.

**Figure 6:**
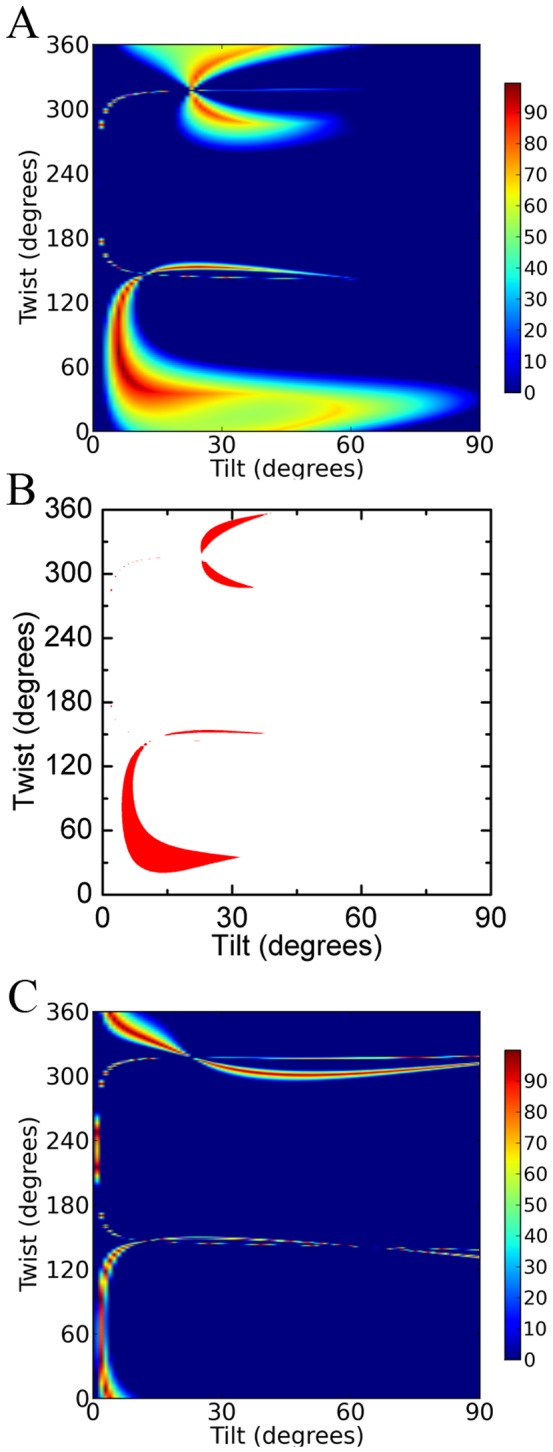
Possible orientations of GRK5_1-531_. (A) The possible orientations of GRK5_1-531_ on POPG lipid bilayers determined by combination of SFG and ATR-FTIR measurements (Figure S6 in File S1) by using the 2ACX crystal structure. (B) The same plot as panel A, but only showing orientations with a score ≥ 70% (red). (C) The possible orientations of GRK5_1-531_ on a 1:1 POPC:PIP_2_ lipid bilayer determined by SFG measurement ($\chi_{zzz}^{\left(2\right)}/\chi_{xxz}^{\left(2\right)}$= 0.87±30%) using the 2ACX crystal structure.

**Figure 7:**
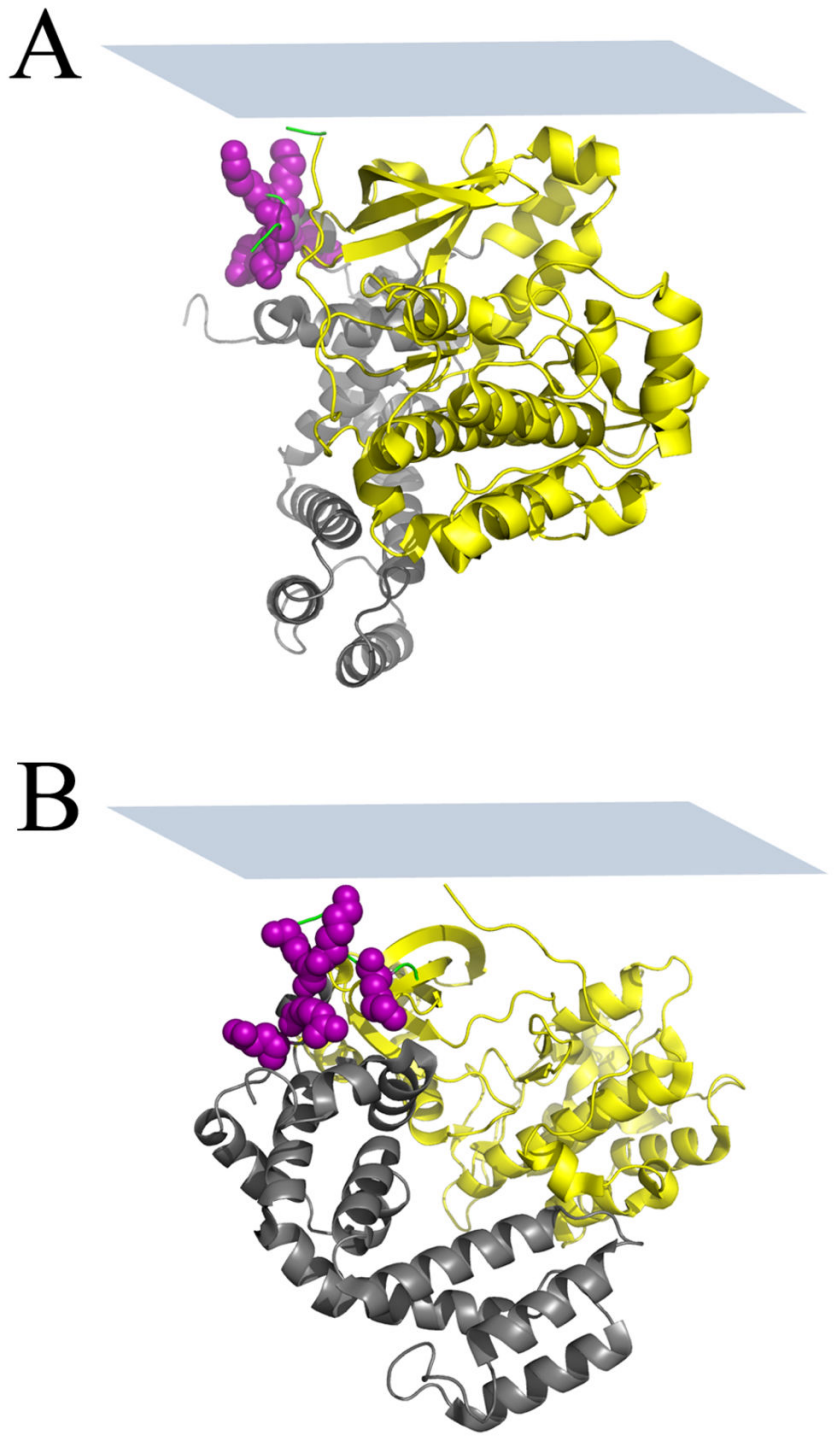
Modeled membrane orientations of GRK5_1-531_. Possible membrane orientations of GRK5_1-531_ on POPG lipid bilayers as determined from SFG and ATR-FTIR experimental measurements by using the 2ACX crystal structure: (A) Twist=40°, Tilt=10°, (B) Twist=300°, Tilt=26°. The plane of the membrane relative to the protein is shown as a blue rectangle.

Once again, the 2ACX model of GRK6 appeared to provide a better fit than 3NYN to describe the orientation ranges of GRK5_1-531_ by either SFG or ATR-FTIR measurements (Figure S4 and S5 in File S1). Figure 6A shows the possible orientations of GRK5_1-531_ by combined SFG and ATR-FTIR measurements by using the crystal structure of 2ACX, and Figure 6B shows the most likely orientation regions of GRK5_1-531_ (with a score > 70%). There are two likely orientation regions (twist, tilt) for GRK5_1-531_: (20-150°, 5–30°) and (280-360°, 25–35°). Two orientations representative orientations from the two ranges shown in Figure 6B are drawn in Figure 7A (40°, 10°) and 7B (300°, 26°). GRK5_1-531_ therefore seems to adopt a similar preferred membrane orientation (Figure 7A and 7B) as full-length GRK5 (Figure 5C and 5D), wherein the N-terminal PIP_2_-binding site is in close proximity with the lipid bilayer. Therefore, the C-terminal amphipathic helix does not seem to have a substantial impact on GRK5 membrane binding or orientation in our experimental system.

PIP_2_ has been proposed to be a specific phospholipid activator of GRK5 [6]. We therefore investigated the orientation of GRK5_1-531_ on a 1:1 POPC:PIP_2_ lipid bilayer. SFG spectra of GRK5_1-531_ on the 1:1 POPC:PIP_2_ (Figure 2G) have similar shape and intensity to those on POPG lipid bilayers (Figure 2F). The measured ratio of $\chi_{zzz}^{\left(2\right)}/\chi_{xxz}^{\left(2\right)}$ for the GRK5_1-531_ α-helical structure at the peak center of 1656 cm^-1^ was 0.93 (Figure 2G), and its likely orientation regions (Figure 6C) are likewise similar to those on the POPG lipid bilayer (Figure 6A). We therefore conclude that GRK5_1-531_ has a similar membrane orientation on POPG and 1:1 POPC:PIP_2_ lipid bilayers.

If the specific orientation of GRK5 and GRK5_1-531_ we observed on POPG is mandated by residues in the N-terminal GRK5 phospholipid binding site, then mutation of these residues should diminish the signal for this specific orientation. We therefore investigated the orientation of GRK5_NT_ on 1:1 POPC:PIP_2_ lipid bilayers. Unlike for GRK5_1-531_, we did not observe any SFG amide I signal from GRK5_NT_ on the 1:1 POPC:PIP_2_ lipid bilayer (Figure 2H). The unpolarized ATR-FTIR spectrum shows that GRK5_NT_ can bind to the 1:1 POPC:PIP_2_ bilayer (Figure S1 in File S1), and thus GRK5_NT_ molecules bind to the membrane but lack a preferred orientation.

We next tested the electrostatic nature of the interactions of GRK5 and GRK5_NT_ with our lipid bilayers by using 150 mM instead of 50 mM NaCl in the buffer. SFG signals from either GRK5 or GRK5_NT_ on the 1:1 POPC:PIP_2_ lipid bilayer were not observed (data not shown), although we detected ATR-FTIR signals from both (Figure S1 in File S1). Thus, the N-terminal phospholipid binding site, which seems to dictate a specific orientation of GRK5, is sensitive to ionic strength. However, this must not be the only such membrane binding determinant because GRK5_1-531_ also binds with random orientations. The extreme N-terminus region of GRK5, which contains conserved hydrophobic residues, does not appear to contribute to membrane binding, as peptides from this region do not seem to bind bilayers as measured by SFG or ATR-FTIR (Bei Ding and Zhan Chen, unpublished data).

## Discussion

In this research, the membrane orientations of GRK5 and GRK5 lacking a C-terminal region containing a conserved amphipathic helix were determined *in situ* using a combination of SFG and ATR-FTIR vibrational spectroscopies. Our results once again show that combined SFG and ATR-FTIR studies narrow down the likely range of orientations exhibited by peripheral membrane proteins. We also found that our spectral data has the ability to distinguish between two distinct structural models, as the 2ACX structure seems to give a better fit to the spectral data, consistent with the 3NYN conformation reflecting that of a GPCR-bound GRK. The deduced membrane orientation indicates that both GRK5 variants assume similar preferred orientations when associated with 100% POPG or 1:1 POPC:PIP_2_ bilayer, with the region that encompasses its PIP_2_ binding site in close proximity to the membrane. GRK5_NT_, which lacks residues in the N-terminal region important for binding PIP_2_, bound to these bilayers but did not exhibit a specific orientation. These results do not rule out an interaction between the C-terminal amphipathic helix of GRK5 and the membrane. However, it seems to be only one of several membrane binding determinants in the protein, such that when this region is truncated it does not have a significant effect on the ability of the enzyme to bind membranes. Our data seems to be in conflict with earlier reports that deletion or perturbation of the C-terminal helix leads to loss of membrane association in cells [7,8]. However, interactions with other proteins likely influence membrane localization in cells, and there are competing processes could readily alter the degree of membrane localization. For example, GRK5 contains a nuclear localization signal [73], and thus loss of one of its membrane binding determinants may shift its equilibrium away from the membrane and towards the membrane.

Reports are conflicting on whether the N-terminal or C-terminal phospholipid binding site of GRK5 is required for binding liposomes *in vitro* [6,7]. This may depend on the exact reaction conditions used (e.g. ionic strength, phospholipid components, wash steps etc.). In our system, we observed at least some degree of membrane binding by all of our GRK5 variants based on unpolarized ATR-FTIR signals. However, a preferred membrane-bound orientation of GRK5 requires the N-terminal phospholipid binding site, as GRK5_NT_ was unable to exhibit this specific orientation. Interaction between the N-terminal binding site and PIP_2_ and or negatively charged POPG was also dependent on ionic strength. The fact that this site could dictate a specific orientation is consistent with the fact that the N-terminal phospholipid binding site is integrated into the GRK5 catalytic core, whereas the C-terminal site is an extension that is joined to the catalytic core by a flexible extension, at least in the inactive state of the enzyme. Thus, although both sites can dictate membrane binding, only the N-terminal site has the capability to mandate a specific orientation that leads to strong SFG or polarized ATR-FTIR signals. Although the C-terminal phospholipid binding site does influence the strength of membrane interaction [7,8], it is not the only element in GRK5 that can drive membrane interactions (albeit random), as GRK5_1-531_, which lacks the C-terminal site, is still membrane associated in our model bilayer system. 

PIP_2_ is known to be important for GPCR phosphorylation by both GRK2 and GRK5 [6]. The orientation of GRK5 bound to POPG bilayers studied here is similar to that determined for GRK2 [5]. In each case, the unique PIP_2_-binding regions of these kinases (the PH domain of GRK2 and the N-terminal region of GRK5) end up positioned close to the expected plane of the inner leaflet of the membrane, suggesting that this orientation is required for efficient coupling to receptors, and that proper electrostatic complementation between the kinase and the membrane is important for function, even though each GRK subfamily uses a unique mechanism to achieve this goal. Thus, GRK2 and GRK4 subfamily members may all adopt a similar orientation when bound to membranes, wherein their PIP_2_ binding sites function to help position the N-terminal region of the GRK in a way that it can efficiently and simultaneously interact with the activated GPCR, and the GRK active site a way that is properly oriented to receive either the third intracellular loop or C-terminal tail of the receptor as a phosphoacceptor. 

Our SFG and ATR-FTIR data, although providing a molecular explanation for why PIP_2_ dramatically activates GRK5 and supporting the idea that PIP_2_-bindng occurs predominantly through the N-terminal phospholipid binding site, cannot discriminate between two possible models for the functional role of the C-terminal amphipathic helix based on biochemical and structural data. In each model, the C-terminus of a GRK4 subfamily member acts as a conformational switch [74]. In the first model, the C-terminal amphipathic helix, in conjunction with adjacent palmitoylation sites, if present, enhances the affinity of the inactive GRK (2ACX-like conformation) for the cell membrane. Activated GPCRs then trigger an allosteric change in the GRK that reconfigures the kinase domain into an active conformation and in which the C-terminal tail of the receptor (residues 532-557) packs against the catalytic core, reinforcing the activated conformation of the enzyme (3NYN-like conformation), and thereby promotes catalysis. This may explain why perturbation of the C-terminal helix leads to defects in auto- and GPCR phosphorylation [8]. However, this model conflicts with the fact that elimination of the C-terminal helix does not seem to affect the phosphorylation of other soluble substrates [7]. Alternatively, the condensed packing of the C-terminus exhibited by the 3NYN structure may reflect the situation when the enzyme is in a soluble form, as GRK5 and GRK6 are both known to shuttle between the membrane, cytoplasm and nucleus depending on signaling conditions [73,75,76]. This would enable other factors, such as phosphorylation, to dramatically affect the affinity of the enzyme for membranes. A similar lipid switch has been proposed for the related enzyme protein kinase A (PKA), whose N-terminal myristoyl group is in equilibrium between membrane and protein-bound states and influenced by phosphorylation at Ser^10^ [77].

## Supporting Information

File S1 (DOCX)
