# Supplementary material for: Membrane Orientation and Binding Determinants of G Protein-Coupled Receptor Kinase 5 as Assessed by Combined Vibrational Spectroscopic Studies
Source: PLoS One. 2013 Nov 22;8(11):e82072. doi: 10.1371/journal.pone.0082072 (PMC3838385; doi:10.1371/journal.pone.0082072)
Supplement: File S1 — (DOCX) [file pone.0082072.s001.docx]

# Supplementary Information

# Membrane Orientation and Binding Determinants of G Protein-Coupled Receptor Kinase 5 as Assessed by Combined Vibrational Spectroscopic Studies

Pei Yang^1^, Alisa Glukhova^2,3^, John J.G. Tesmer^2*^, Zhan Chen^1*^

^1^ Department of Chemistry, University of Michigan, 930 North University Avenue, Ann Arbor, Michigan 48109, USA

^2^ Departments of Pharmacology and Biological Chemistry, Life Sciences Institute, University of Michigan, Ann Arbor, Michigan 48109, USA

^3^ Program in Chemical Biology, University of Michigan, USA

*Email: zhanc@umich.edu; tesmerjj@umich.edu

**1. Unpolarized ATR-FTIR spectra**

Unpolarized ATR-FTIR was used to compare the surface coverage of GRK5 or GRK5_1-531_ on 9:1 POPC:POPG and pure POPG lipid bilayers. The ATR-FTIR signal of protein is proportional to the number of protein molecules binding to the lipid bilayer surface. Results showed that the ATR-FTIR signals of GRK5 and GRK5_1-531_ are similar on different lipid bilayers, indicating that the surface coverages of GRK5 (Figure S1A) and GRK5_1-531_ (Figure S1B) are not very different on either 9:1 POPC:POPG or POPG lipid bilayers. Supplementary Figure S1C shows the unpolarized ATR-FTIR spectra from GRK5_NT_ (50 and 150 mM NaCl in buffer) and GRK5 (150 mM NaCl in buffer) on a 1:1 POPC:PIP_2_ lipid bilayer, showing that both GRK5_NT_ and GRK5 can bind to the 1:1 POPC:PIP_2_ bilayer with a similar surface coverage.


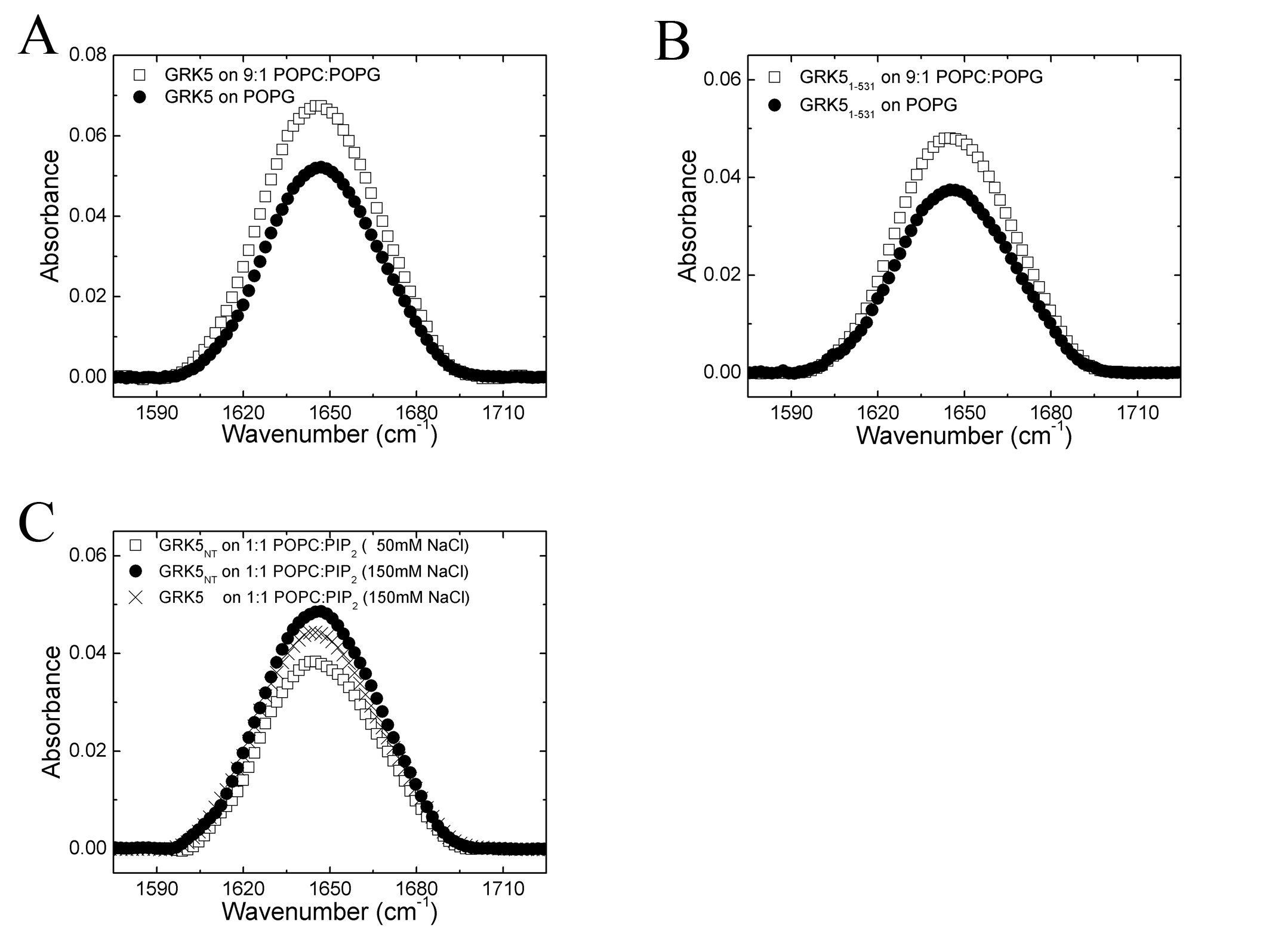


**Figure S1. The unpolarized ATR-FTIR spectra**. Unpolarized ATR-FTIR spectra of 336 nM (A) GRK5 and (B) GRK5_1-531_ on 9:1 POPC:POPG (open square dots) and pure POPG (solid circles) lipid bilayer. (C) Unpolarized ATR-FTIR spectra of 336 nM GRK5_NT_ (50 and 150 mM NaCl in buffer) and GRK5 (150 mM NaCl in buffer) on 1:1 POPC:PIP_2_ lipid bilayer.

**2. SFG and ATR-FTIR orientation analysis**

We have previously reported the determination of membrane orientation of G-protein by using SFG and ATR-FTIR methods [1,2]. Computer programs were developed to facilitate protein orientation analysis by calculating the SFG signal ratios ($\chi_{zzz}^{(2)}/\chi_{xxz}^{(2)}$) and ATR-FTIR dichroic ratio (R^ATR^) at any orientation of a given crystal structure relative to the lipid bilayer [1,2].


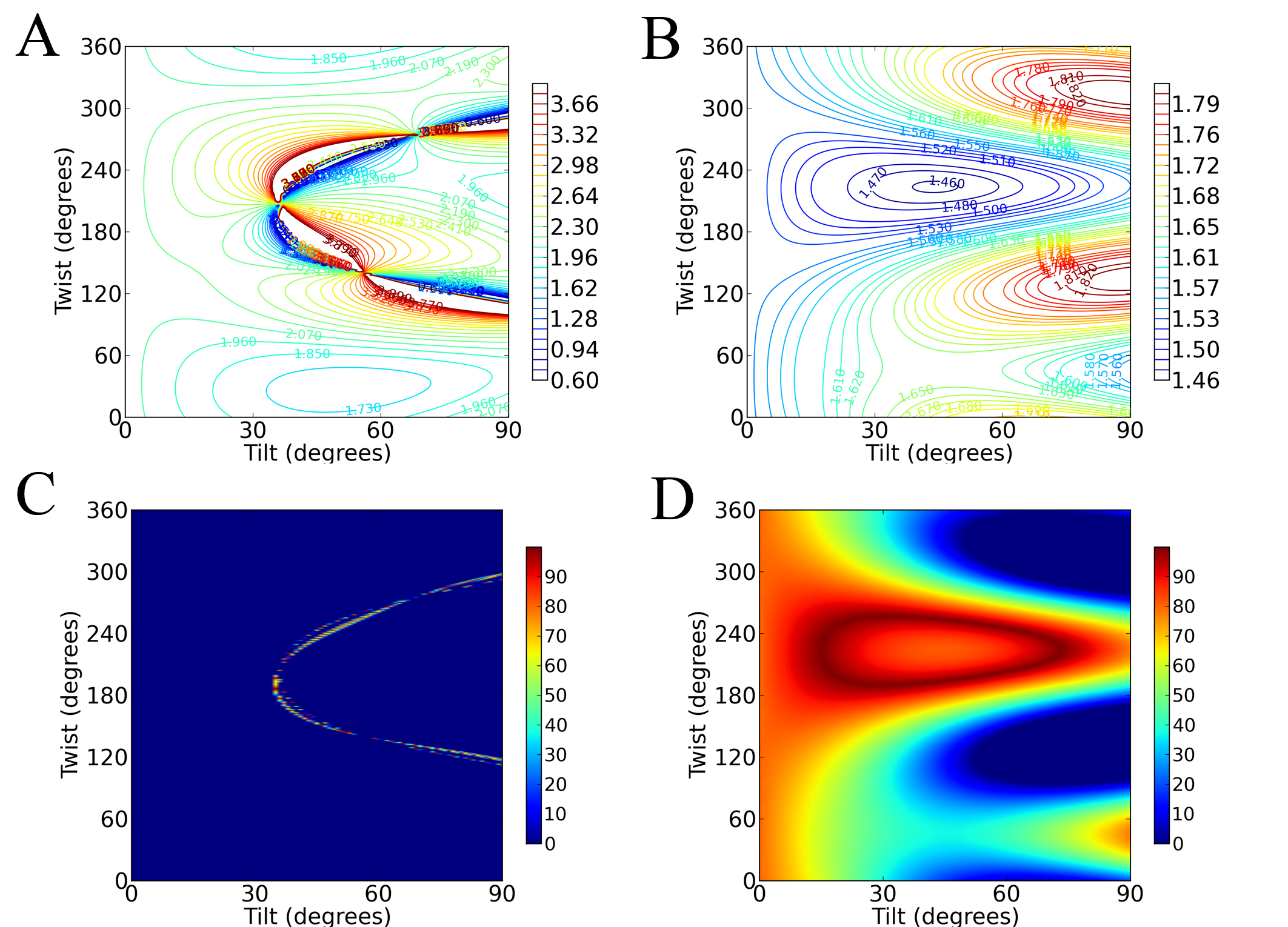


**Figure S2. Possible orientation of GRK5 using the 3NYN crystal structure.** Contour plots showing the (A) calculated SFG ratio ($\chi_{zzz}^{(2)}/\chi_{xxz}^{(2)}$) and (B) calculated ATR-FTIR dichroic ratio R^ATR^ of GRK5 for all unique twist (ψ) and tilt (θ) angles by using the 3NYN crystal structure. The possible orientations of GRK5 on POPG lipid bilayers determined by (C) SFG measurement ($\chi_{zzz}^{(2)}/\chi_{xxz}^{(2)}$=0.93±30%), and (D) ATR-FTIR measurement (dichroic ratio R^ATR^ =1.5±15%). The effect of experimental errors (such as uncertainty in the Fresnel coefficients) is accounted for using a coloring scheme based on how well the calculated and experimentally measured quantities agree for each possible orientation, within specified error bars (1,2). If the calculated $\chi_{zzz}^{(2)}/\chi_{xxz}^{(2)}$ ratio does not match the experimental value within error bars, a score of 0 was assigned. A score of 100% indicates an exact match.


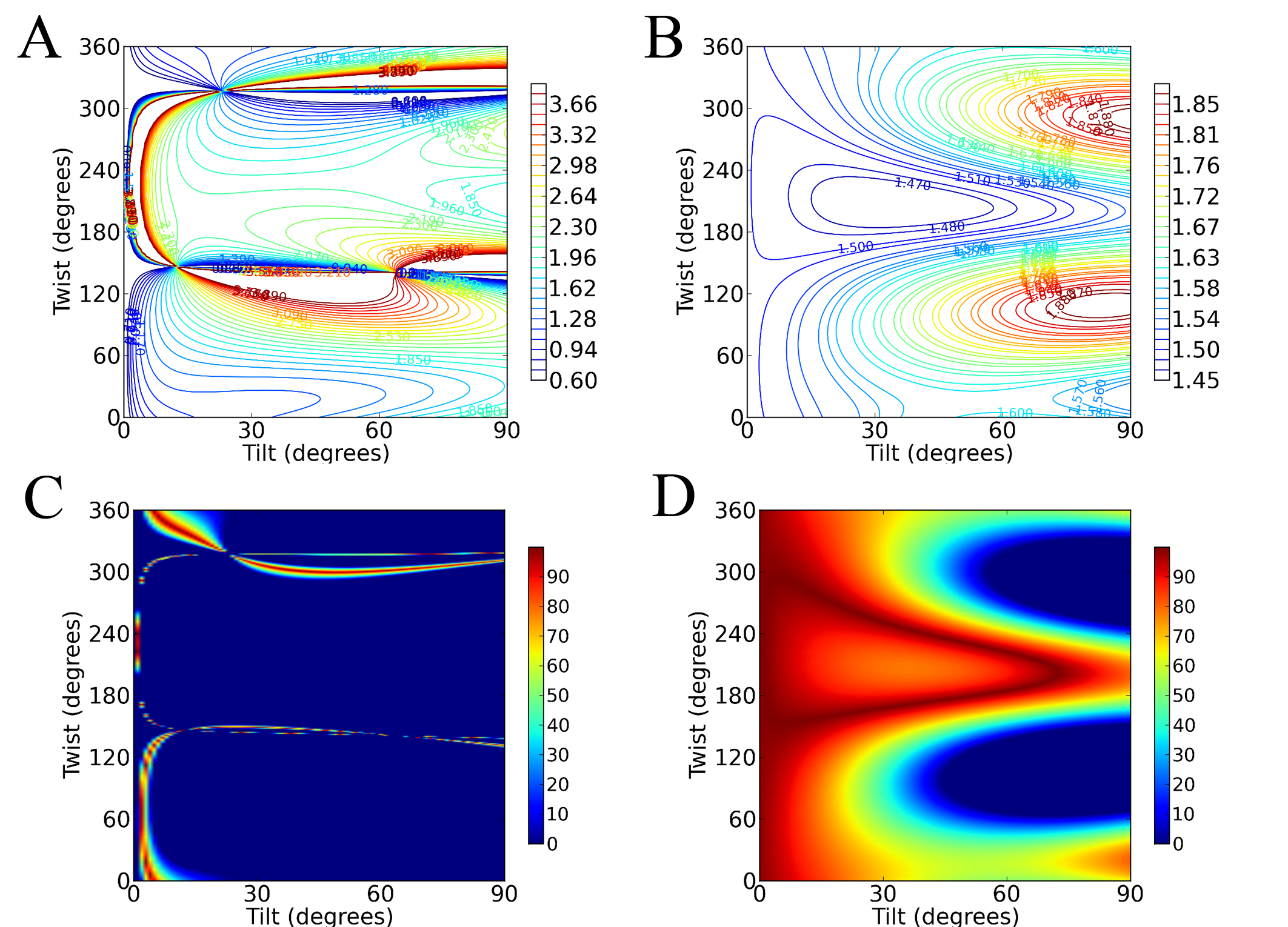


**Figure S3. Possible orientation of GRK5 using the 2ACX crystal structure.** Contour plots showing the (A) calculated SFG ratio $\chi_{zzz}^{(2)}/\chi_{xxz}^{(2)}$ and (B) calculated ATR-FTIR dichroic ratio R^ATR^ of GRK5 for all unique combinations of the twist (ψ) and tilt (θ) angles using the 2ACX crystal structure. Orientation of GRK5 on a POPG lipid bilayer at which the calculated values best match experimentally measured value for (C) SFG ratio of $\chi_{zzz}^{(2)}/\chi_{xxz}^{(2)}$ (0.93±30%), and (D) the ATR-FTIR dichroic ratio R^ATR^ (1.5±15%).


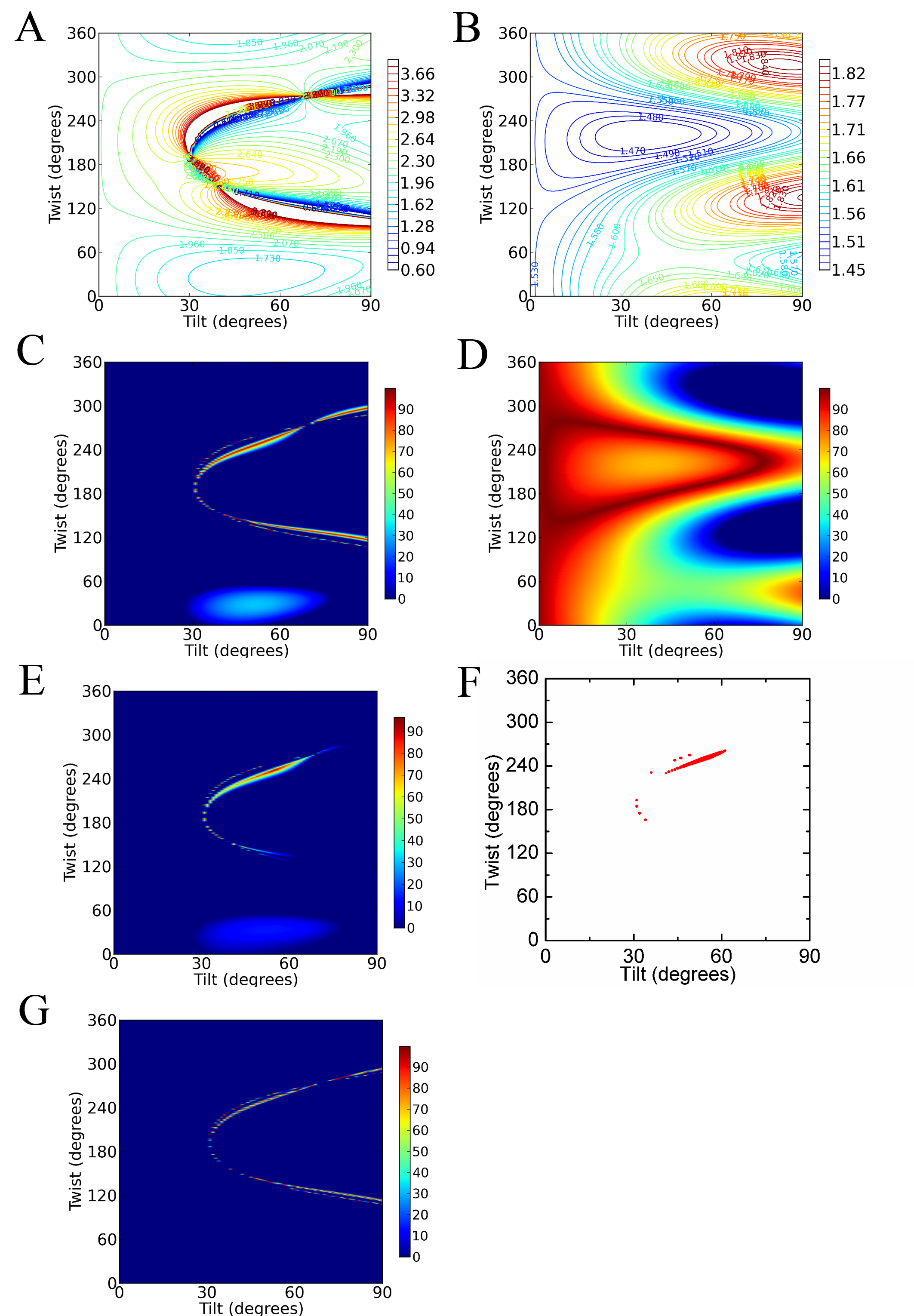


**Figure S4. Possible orientation of GRK5_1-531_ using the 3NYN crystal structure.** Contour plots showing the (A) calculated SFG ratio $\chi_{zzz}^{(2)}/\chi_{xxz}^{(2)}$ and (B) calculated ATR-FTIR dichroic ratio R^ATR^ of GRK5_1-531_ for all unique combinations of the twist (ψ) and tilt (θ) angles by using the 3NYN crystal structure with C-terminal residues beyond residue 531 deleted. Orientation of GRK5_1-531_ on POPG lipid bilayers at which the calculated values best match experimentally measured value for (C) SFG ratio of $\chi_{zzz}^{(2)}/\chi_{xxz}^{(2)}$ (1.35±30%), (D) the ATR-FTIR dichroic ratio R^ATR^ (1.52±15%), and (E) combination of SFG and ATR-FTIR measurements. (F) The same plot as panel E, but only showing orientation areas with a score ≥ 70% (red). There are two likely orientation ranges (twist, tilt) for GRK5_1-531_: (170-200˚, 30-35˚) and (230-260˚, 45-60˚). (G) The possible orientations of GRK5_1-531_ on a 1:1 POPC:PIP_2_ lipid bilayer determined by SFG measurement ($\chi_{zzz}^{(2)}/\chi_{xxz}^{(2)}$= 0.87±30%).


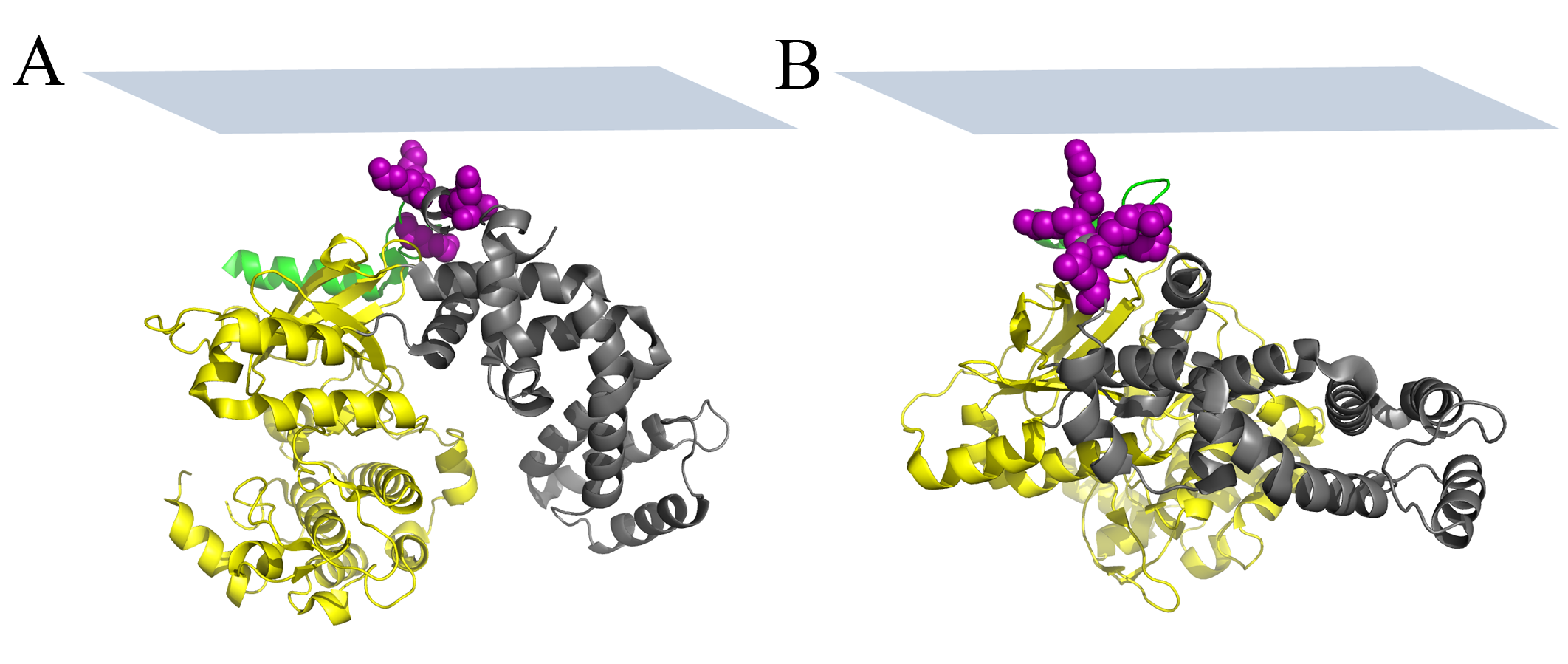


**Figure S5. Possible membrane orientations of GRK5_1-531_.** Possible membrane orientations of GRK5_1-531_ on POPG lipid bilayers as determined from SFG and ATR-FTIR experimental measurements using the crystal structure of 3NYN (C-terminus structure was deleted): (A) twist=190˚, tilt=35˚, (B) twist=245˚, tilt=50˚. The plane of the membrane relative to the protein is shown as a blue rectangle.


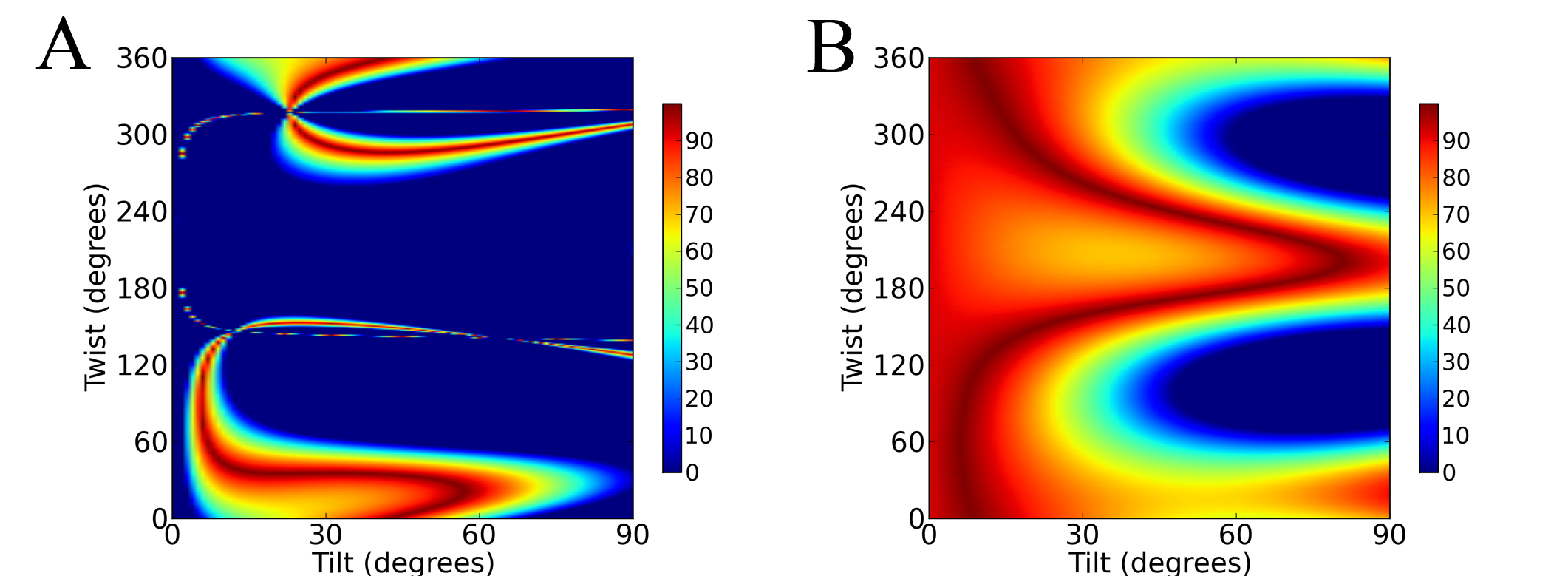


**Figure S6. Possible orientation of GRK5_1-531_ using the crystal structure of 2ACX.** Calculated possible orientation of GRK5_1-531_ on a POPG lipid bilayer by using the crystal structure of 2ACX for (A) SFG ratio of $\chi_{zzz}^{(2)}/\chi_{xxz}^{(2)}$ (1.35±30%), and (B) the ATR-FTIR dichroic ratio R^ATR^ (1.52±15%).

**SUPPLEMENTARY REFERENCES:**

1. Boughton AP, Yang P, Tesmer VM, Ding B, Tesmer JJ, et al. (2011) Heterotrimeric G protein β1γ2 Subunits Change Orientation upon Complex Formation with G Protein-coupled Receptor Kinase 2 (GRK2) on a Model Membrane. Proc Natl Acad Sci U S A 108: E667-E673.
2. Yang P, Boughton AP, Homan KT, Tesmer JJG, Chen Z (2013) Membrane Orientation of Gαiβ1γ2 and Gβ1γ2 Determined via Combined Vibrational Spectroscopic Studies. J Am Chem Soc. 135: 5044-5051.
